# Identifying the supportive care needs of people affected by non-muscle invasive bladder cancer: An integrative systematic review

**DOI:** 10.1007/s11764-024-01558-7

**Published:** 2024-03-23

**Authors:** Kathryn Schubach, Theo Niyonsenga, Murray Turner, Catherine Paterson

**Affiliations:** 1 Faculty of Health, University of Canberra, Bruce, ACT Australia; 2 Caring Futures Institute, Flinders University, Adelaide, Australia; 3 Central Adelaide Local Health Network, Adelaide, Australia; 4 Robert Gordon University, Aberdeen, Scotland UK

**Keywords:** Non-muscle invasive bladder cancer, Supportive care, Patient experience

## Abstract

**Purpose:**

To understand supportive care needs among people with non-muscle invasive bladder cancer (NMIBC).

**Methods:**

An integrative systematic review was reported using the Preformed Reporting Items for Systematic Review and Meta-analyses (PRISMA) guidelines. Seven electronic databases were searched for relevant studies, including all quantitative, qualitative, and mixed methods studies, irrespective of research design. The review process was managed by Covidence systematic review software. Two reviewer authors independently performed data extraction using eligibility criteria. Quality appraisal was conducted, and a narrative synthesis was performed.

**Results:**

A total of 1129 articles were screened, of which 21 studies met the inclusion criteria. The findings revealed that the frequency of supportive care needs reported by NMIBC participants included psychological/emotional (16/21:76%), physical (16/21:76%), practical (8/21:38%), interpersonal/intimacy (7/21:33%), family-related (7/21:33%), health system/information (5/21:23%), social (4/21:19%), patient-clinician communication (3/21:14%), spiritual (1/21:5%) and daily needs (1/21:5%).

**Conclusion:**

People affected by NMIBC experience anxiety, depression, uncertainty, and fear of recurrence. The physical symptoms reported included urinary issues, pain, sleeping disorders and fatigue. These supportive care needs persist throughout the participants' treatment trajectory and can impact their quality of life.

**Implications for Cancer Survivors:**

Identifying supportive care needs within the NMIBC population will help inform future interventions to provide patient-centred care to promote optimal well-being and self-efficacy for people diagnosed with NMIBC.

**Supplementary Information:**

The online version contains supplementary material available at 10.1007/s11764-024-01558-7.

## Introduction

Bladder cancer is the tenth most prominent cancer diagnosis globally and remains the most expensive cancer to treat [1], with approximately 550,000 individuals diagnosed yearly. The highest incidence rates occur in Europe and North America [2] and it is the eleventh-ranked cancer diagnosis in Australia. Most bladder tumours (75–80%) present as non-muscle invasive bladder cancer (NMIBC) [3–6].

The known causal risk factors of bladder cancer include smoking and occupational exposure to amines and other chemicals [7, 8]. Other causes that have been shown to increase the risk of bladder cancer include chronic urinary tract infections, previous radiotherapy to the pelvis, exposure to cyclophosphamide, and exposure to contaminated drinking water by parasites such as *Schistosoma haematobium* [2, 9].

The treatment for NMIBC involves a complete surgical transurethral resection of the bladder tumour. Further treatment decisions are then initiated based on the histology results of the resected tumour. Cancer staging depends on the pathological grading and the depth of the tumour [10]. Tumours are classified as NMIBC when there is no evidence of tumour invasion into the lamina propria. The categories of NMIBC include (1) Ta- (non-invasive papillary tumour), (2) Tis-(carcinoma in situ) and (3) T1- (tumour invades subepithelial connective tissue) [11, 12].

Treatment involves regular invasive surveillance and either intravesical immunotherapy or chemotherapy. High-grade tumours are treated with Bacillus Calmette-Guerin (BCG) with induction and maintenance courses or in combination with intravesical chemotherapy such as Mitomycin C [5, 6]. A prospective study by Grossman et al. (2022) found that the 5-year risk of recurrence or progression was high at 83% in patients with high-risk NMIBC [13]. The risk of NMIBC becoming muscle-invasive has been reported to be 20–25% during the patient’s lifetime [14]. Consequently, the burden of treatment regimens, coupled with frequent and invasive surveillance protocols, means that most patients are at risk of reduced quality of life, psychological challenges, and a range of unmet supportive care needs despite routine clinical follow-ups with healthcare professionals [5, 15, 16].

It has been suggested that more research focused on the quality of life and supportive care needs among people living with NMIBC [4, 5, 15], particularly for patients with high-risk NMIBC clinical features. Evidence has underscored that the bladder cancer research focus has focused on predominately muscle-invasive bladder cancer [17]. Furthermore, a systematic review [18] evidenced unmet supportive care needs exclusive to people affected by muscle-invasive bladder cancer, which therefore provides no insight into the needs of those affected by NMIBC.

A synthesis of current knowledge [19–21] has revealed that patients have reported a decreased quality of life following their initial diagnosis of NMIBC cancer. A decrease in quality of life is associated with distressing side effects of treatment and psychological issues such as anxiety, depression and uncertainty. Patients also experienced embarrassment due to the invasive nature of their surveillance procedures (i.e. cystoscopy procedures) [21].

Supportive care needs have been defined as the patient’s request for both general support or an identified problem prioritised by the individual when diagnosed or treated for cancer [19, 22]. Supportive care needs can occur from diagnosis through the treatment phase and into either the survivorship or palliative phases of the illness [21, 23]. Supportive care is classified into several domains: physical, emotional/psychological, cognitive, patient-clinician, health system/informational, spiritual, daily living, interpersonal, intimacy, practical and social needs [24, 25]. Timely identification of patients’ supportive care needs is paramount to ensure that patients receive optimised care to enhance health outcomes by addressing what matters most to cancer patients [23].

Several studies [19–21] have identified the relationship between unmet needs and reduced quality of life. Unmet supportive care needs may lead to emotional distress and higher symptom distress scores and can negatively impact patients’ coping abilities throughout their care trajectory [26–29]. These effects contribute to a diminished quality of life [20, 30, 31]. To date, the evidence has yet to be critically synthesised to understand the supportive care needs among people living with NMIBC specifically. Current knowledge in this area is timely and important to inform clinical practice, any requirements for service re-design, and future research directions.

Therefore, this integrative systematic review aimed to address the following research questions:

1. What are the supportive care needs among people affected by NMIBC cancer?
2. What are the frequently reported domains of supportive care needs among people affected by NMIBC?

## Methods

This review was reported according to the Preferred Reporting Items for Systematic Reviews and Meta‐Analyses (PRISMA) guidelines [32]. A review protocol was developed and registered with PROSPERO (CRD 42022332137).

### Search strategy

The following electronic databases and register were searched by an expert systematic review librarian: APA PsycINFO, CINAHL, Cochrane Library (DSR and CENTRAL), MEDLINE, Scopus, and Web of Science Core Collection, date cut-off from inception to December 2022. See Supplementary Table 1 for the full search strategy.

### Eligibility criteria

#### Inclusion criteria

All studies were included if they investigated the supportive care needs among adults (> 18 years) diagnosed with NMIBC, including all qualitative, quantitative and mixed methods. Patients in mixed cancer groups were included only when separate subgroup analyses were reported for NMIBC participants.

#### Exclusion criteria

Studies where supportive care needs were not explicitly reported or conducted were excluded.

### Study collection and data extraction

#### Screening process

All articles identified were imported into Endnote referencing software and exported to Covidence Systematic Review software (Covidence© 2020, Version 1517, Melbourne, Australia) for the removal of duplicates and the study selection process. The articles were screened, and two reviewers applied the inclusion criterion to all titles and abstracts and any conflicts were resolved by discussion. Reviewers then assessed the full-text articles, and disagreements were resolved through discussion. The study selection process was described using the PRISMA diagram [32]. Full-text studies that did not meet inclusion criteria were excluded with reasons.

#### Data extraction

One reviewer (KS) extracted study data, and a second reviewer (CP) checked for quality and accuracy. A data extraction table was developed and piloted in a sample of studies prior to data extracting for all of the studies. The data extraction table contained information about the participants' clinical and demographic characteristics, countries and institutions where data was collected, setting, sample size, study design, reports of supportive care needs, and the number of participants included in the studies. A second data extraction table was used for the qualitative data.

### Quality assessment

The methodological quality and evaluation of the studies were assessed using the mixed methods appraisal tool (MMAT) [33]. The MMAT tool was selected for its versatility when assessing different study designs in this integrative review. The MMAT tool enabled critical assessment of quantitative, qualitative and mixed-method studies included in this review. All domains were assessed and rated against “no”, “yes”, and “unclear”. Methodological quality assessment was performed by one reviewer, and quality was checked by a second reviewer.

### Data synthesis

This review used a narrative synthesis and tabulation of primary research studies to identify the supportive care needs of NMIBC population. The narrative synthesis included the following steps: data reduction (subgroup classification based on levels of evidence and research questions), data comparison (an iterative process of making comparisons and identifying relationships) and conclusion substantiation [34]. This approach has been used in several cancer systematic reviews [25, 31, 35] identifying supportive care needs among various cancer groups.

### Operational definition of domains of need

Supportive care needs were categorised into eleven primary domains of need based on current literature, the seminal work of Fitch (2008), and clinical expertise. Specifically, the domains include physical, psychosocial/emotional, family-related, social, interpersonal/intimacy, practical, daily living, spiritual/existential, health system/information, patient/clinician communication, and cognitive needs [24, 25, 31] (see Fig. 2).

## Findings

**Fig. 1:**
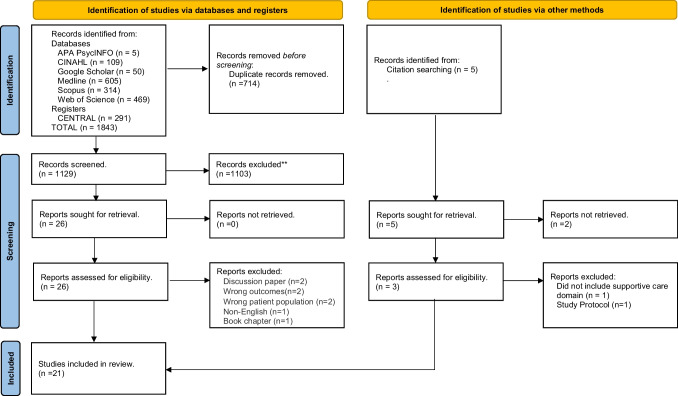
PRISMA diagram [32]

Figure 1 provides an overview of the screening and selection process. A total of 21 studies were included and met the inclusion criteria, and complete data extraction and quality assessment are presented in Supplementary Table 2.

### Study characteristics

A total of 21 studies were included in this integrative review. See Table 1 for an overview of the studies. The various study designs that included the types of studies presented in this systematic review comprised: qualitative *n* = 2 [36, 37], quantitative *n* = 16 [26, 27, 29, 38–50] and *n* = 3 mixed methods [28, 51, 52]. This systematic review comprised 3654 participants: *n* = 2918 males, female *n* = 736. The sample sizes ranged from 6 to 868 participants. The studies included representation from several countries, including the USA, the UK, the Netherlands, China, Greece, Korea, and Japan. Noteworthy, there is no representation from the Australian or New Zealand populations. The median age of the patients was 67 years (min. = 46, max. = 89). Their clinical status included pathological grading of tumours as Ta- pT1 and carcinoma in situ. Eleven studies represented patients treated with BCG or intravesical chemotherapy [27, 29, 36, 38–40, 43, 44, 46, 48, 49].

**Table 1:** Overview of the included studies

Author and Year Country	Purpose	Setting	Sample size	Participants	Sampling	Response rate	Attrition	Design	Time points	Data collection tools
Alcorn et al., 2020. United Kingdom	To explore what influences patients’ experiences of withdrawal from BCG treatment	National Health Service Trust (Metropolitan) Northern England	*N*= 6	**Clinical** Treatment- Number of BCG cycles **1** *N* = 1, **2** *N* = 3, **3** *N* = 2 **Cancer Grade & Stage –** G3 pTa = N1, G3 pT1 *N* = 2, G3 pT1 & CIS *N* = 2, CIS-*N* = 1 **Demographics Age**- Male 57,61,78, **Age-** Females 60,63,79 **Gender:** Male *N* = 3, Female *N* = 3, **Marital status**: Not Reported **Smokers**: Not reported **Employment**: Not Reported **Education:** Not Reported	Non- Probability purposive sampling	Not reported	N/A	Qualitative	1	**Questionnaires:** Demographics: Unspecified questionnaire **Qualitative:** Semi-structured interviews
Brisbane et al., 2018 USA	To investigate the impact of health-related quality of life with the diagnosis and treatment of NMIBC	Using Surveillance, Epidemiology, and End Results -Medicare Health Outcome Survey (SEER-MHOS) database	*N* = 325 Controls *N* = 1625 1:5	**Clinical**: **Treatment**-Not Reported NMIBC-*N*= 325 Non-cancer Control (NCC) *N*= 1625 **Cancer Grade & Stage**: -Ta *N* = 211, T1 *N* = 88, CIS *N* = 26 **Demographics: Age:** SD NMIBC- 75.6 (6.6), NCC-73.8 **Gender**: Male NMIBC *N* = 235, Female *N* = 90. NCC- Males *N* = 1179, Female *N* = 446 **Marital status**: NMIBC-Married *N* = 202, Divorced *N* = 35, Widowed *N* = 65, Other *N* = 23. NCC- Married *N* = 995, Divorced *N* = 186, Widowed *N* = 324, Other *N* = 120 **Smokers**: NMIBC- YES *N* = 45, No *N* = 280.NCC-YES *N*= 236, NO *N* = 1389 **Employment**: Not Reported **Education:** NMIBC- High School Graduate *N* = 139. NCC High School Graduate *N* = 640	Convenience	Not Reported	Not Reported	Quantitative	1	**Questionnaires:** Demographics: Unspecified questionnaire. Quality of life: Use of a combination. The 36-Item Short Form Health Survey (SF-36), and The Veterans RAND 12-Item Health Survey (VR 12). Health domain: The Medicare Health Outcomes Survey (MHOS), Urinary function assessment: The Medicare Health Outcomes Survey includes Four questions assessing urinary incontinence
Catto et al., 2021 United Kingdom	To analyse at a population level the health-related QOL of individuals diagnosed with bladder cancer within 10 years	National Health Services (NHS) Hospitals in Yorkshire, Humber, North Derbyshire, South Tees region of London	*N*= 868 NMIBC 48% (1796 total)	**Clinical**: **Treatment**- TURBT-*N*= 306, TURBT/BCG/MMC *N*= 582 RC-*N*= 405, RC/other treatments *N*= 299, Radical RT *N*= 155 NMIBC cohort 43% of respondents had NMIBC. No stage recorded 39% **Cancer Grade & Stage**: at diagnosis Stage 1 *N*= 528, unknown *N*= 220 **Demographics: Age**: TURBT-Median age at diagnosis 71 (64–78) Median age at survey 78 (71–84), TURBT-BCG/MMC at Median age at diagnosis 69 (63–75), Median at survey 76 (70–82) **Gender**: Male *N*= 684, Female *N*= 184 **Marital status**: Not reported **Smokers**: Not reported **Employment:** Not reported **Education:** Not reported	Convenience	53%	Not reported	Quantitative	1	**Questionnaires:** Demographics: Unspecified questionnaire. Quality of life: five-level EQ-5D (EQ-5D-5L), European Organisation for the Research and Treatment of Cancer- Quality of Life -Superficial Bladder Cancer-24 (EORTC QLQ-BLS-24), European Organisation for the Research and Treatment of Cancer- Quality of Life (QLQ-C30),
Chung et al., 2019 Canada	To investigate the subset of NMIBC patients in relation to QOL, informational and supportive needs	Princess Margaret Cancer Centre (Genitourinary Clinic) The Ottawa Hospital (Urology Clinic) Bladder Cancer Canada (Support	NMIBC *N* = 337 Total Participants *N* = 586	**Clinical**: NMIBC *N* = 337–57% **Treatment**- Surgery only *N* = 170, Chemotherapy only *N* = 16, BCG Only *N* = 33, RT-*N*= 3, Surgery + Chemo *N* = 108, Surgery + BCG *N* = 261, Surgery + RT *N* = 35 **Cancer Grade & stage**: Ta, CIS, T1 *N* = 324 **Demographics: Age**: *Not reported (NMIBC-separately) **Gender**: *Not reported **Marital status**: * Not reported **Smokers**: *Not reported Employment: *Not reported Education: * Not reported	Convenience	52% (total)	Not reported	Quantitative	1	**Questionnaires:** Demographics: Unspecified questionnaire. Quality of Life: Bladder utility Symptom Scale (BUSS). Informational needs: internally designed non-validated questionnaire. Supportive care needs: Cancer Survivorship Unmet Needs tool (CaSUN)
Garg et al., 2018 USA	To describe the experience of NMIBC patients, define care priorities and identify needs for improvement through the cancer continuum	GHS Cancer Registry (Geisinger Rural Community setting)	*N*= 20	**Clinical**: NMIBC **Treatment-** Not reported **Cancer Grade & Stage**: < T2 **Demographics: Age:** 46–85 **Gender**: Male *N* = 16, Female *N* = 4 **Marital status**: Not reported **Smokers**: Not reported **Employment:** Not reported **Education:** Not reported	Randomly selected	87 patients approached 20 accepted	Not reported	Qualitative	1	**Questionnaires:** Demographics: Unspecified questionnaire **Qualitative:** Semi-structured interviews, 3 Focus groups (90 min) (7 participants in each group) Semi-structured focus group guide was developed and included how they found out they had bladder cancer, decision management, goals of care, and how their life has changed since their diagnosis
Jung et al., 2021 USA	To analyse the prevalence of PTSD symptoms and identify the predictive factors associated with non-muscle invasive bladder cancer survivors	North Carolina Central Cancer Registry	*N*= 376	**Clinical**: **Treatment**- TURBT-*N*= 276, Intravesical Immunotherapy *N*= 163, Intravesical Chemotherapy *N* = 136, Removal of bladder *N* = 21, radiation therapy *N* = 3, Other *N* = 14, No response *N* = 11 Not in treatment *N* = 299, Receiving Treatment *N* = 71 No response *N* = 6 Current cystoscopy frequency 0–3 months *N*= 109, 4–6 months *N* = 161, 7–11 months *N* = 18 1 year *N*= 53, 2 Year *N* = 3, once *N*= 10, no response given *N* = 2218 Current NMIBC Status Cured *N* = 243 Not cured *N* = 37, Don’t Know *N* = 88, no response *N*= 8 Recurrence/Progression of NMIBC history Recurrence Yes *N* = 153, Progression Yes *N* = 8, No *N* = 207, No response *N*= 8 **Cancer Grade & Stage**: (At diagnosis) Ta *N* = 250, Tis *N* = 24, T1 *N* = 102 **Demographics: Age**: At diagnosis Mean 68.3 SD (9.2) 34–89, Age at Study enrolment Mean 72.2 SD (9.2) 39–94 **Gender**: Male *N* = 272, Female *N* = 104 **Marital status**: Married /living with partner *N* = 284, Never married/Divorced/Widowed/ separated *N* = 80 no response given *N* = 12 **Smokers**: **Smoking History** (at least 100 cigarettes/5 packs) in entire life Yes *N* = 268 No *N*= 103 No response *N*= 5 Current Smoking Yes *N* = 40, No *N* = 329, No response *N* = 7 **Employment:** Employed *N* = 71, Unemployed *N* = 20, Retired *N* = 274, No response *N* = 11 **Education:** High school, graduate or less *N* = 89, Some college or technical school *N* = 210, Post graduate *N* = 65, No response N-12	Randomly selected	23.6%	Not reported	Quantitative	1	**Questionnaires:** Demographics: Unspecified questionnaire. Health problems: Self-Administered Comorbidity Questionnaire (SCQ). Psychosocial Characteristics: Medical Outcomes Study (MOS) Social Support Survey. Cognition: Patient-Reported Outcomes Measurement System (PROMIS)-Applied Cognition-Abilities Short Form v 1.0, PROMIS Applied Cognition- General Concerns Short Form v1.0. Post-Traumatic Stress Symptoms: Diagnostic and Statistical Manual of Mental Disorders (DSM-5) (PCL)-5) instructions modified to reflect bladder cancer diagnosis and treatment
Jung et al., 2022 USA	To examine the relationship among uncertainty, PTSD symptoms and QOL in NMIBC patients	North Carolina Central Cancer Registry	*N*= 376	**Clinical**: **Treatment**- TURBT-*N*= 276, Intravesical Immunotherapy *N* = 163, Intravesical Chemotherapy *N* = 136, Removal of bladder *N* = 21, radiation therapy *N* = 3, Other *N* = 14, No response *N* = 11 Not in treatment *N* = 299, Receiving Treatment *N* = 71 No response *N* = 6 Current cystoscopy frequency 0–3 months *N* = 109, 4–6 months *N* = 161, 7–11 months *N* = 18 1-year *N* = 53, 2 Year *N* = 3, once *N* = 10, no response given *N* = 2218 Current NMIBC Status Cured *N* = 243 Not cured *N* = 37, Don’t Know *N* = 88, no response *N* = 8 Recurrence/Progression of NMIBC history Recurrence Yes *N* = 153, Progression Yes *N* = 8, No *N* = 207, No response *N*= 8 **Cancer Grade & Stage**: (At diagnosis) Ta *N* = 250, Tis *N* = 24, T1 *N* = 102 **Demographics: Age**: At diagnosis Mean 68.3 SD (9.2) 34–89, Age at Study enrolment Mean 72.2 SD (9.2) 39–94 **Gender**: Male *N* = 272, Female *N*= 104 **Marital status**: Married /living with partner *N* = 284, Never married/Divorced/Widowed/ separated *N* = 80 no response given *N* = 12 **Smokers**: **Smoking History** (at least 100 cigarettes/5 packs) in entire life Yes *N* = 268 No *N* = 103 No response *N* = 5 Current Smoking Yes *N* = 40, No *N* = 329, No response *N* = 7 **Employment:** Employed *N* = 71, Unemployed *N* = 20, Retired *N* = 274, No response *N* = 11 **Education:** High school, graduate or less *N* = 89, Some college or technical school *N* = 210, Post graduate *N*= 65, No response N-12	Convenience	Not reported	Not reported	Quantitative	1	**Questionnaires:** Demographics: Unspecified questionnaire. Quality of Life: European Organisation for the Research and Treatment of Cancer- Quality of Life (QLQ-C30). Social support: Medical Outcomes Study-Social Support Survey 9MOS-SS). Cognition: Patient-Reported Outcomes Measurement System PROMIS Applied Cognition-Abilities Short Form. PROMIS -General Concerns Short Form. Uncertainty: The Mishel Uncertainty of the cancer survivors (MUIS-S). Post-traumatic distress disorders symptoms (PTSD, PTSS) Diagnostic and Statistical Manual of Mental disorders DSM-5 (PCL5)
Koo et al., 2017 USA	To explore the physical and psychosocial factors that affect patients’ experiences and perceptions during bladder cancer surveillance for NMIBC	Veteran Affairs Medical Center White River Junction Vermont	*N*= 12	**Clinical**: The mean time since the first bladder cancer diagnosis is 6.5 years **Treatment**-Number of cystoscopies performed mean 6.5 IQR (4–10) TURB-1–7, unknown TURB *N* = 3 **Cancer Grade & Stage**: Ta-*N*= 5, High grade-*N*= 4, TIS *N* = 1, Unknown *n* = 2 **Demographics: Age**: Not reported **Gender**: Male *N* = 10, Female *N* = 2 **Marital status**: Not reported **Smokers**: Not reported **Employment**: Not reported **Education:** Not reported	Convenience	43%	Not reported	Mixed Methods	1	**Questionnaires:** Demographics: Unspecified questionnaire. Psychological: Psychological consequences of a screening questionnaire (PCQ). Satisfaction: Customer Satisfaction Survey (CSS) (adapted for study) **Qualitative:** One 90-min semi-structured focus group (3–5 participants)
Kowalkowski et al., 2014 USA	To determine the impact of sexual dysfunction on NMIBC survivors	Study 1 Veterans from large urban VA tumour registry lists were contacted and screened using opt-out letters Study 2 Recruited by posting ads on bladder cancer survivorship websites, potential respondents were asked to contact study	Study 1-*N*= 177 Study 2 *N*= 26	**Clinical:** Diagnosed NMIBC within 4 Years **Treatment** – not reported **Cancer Grade & Stage**: **Study 1** Tumour stage, Ta *N*= 37, TIS *N*= 15, T1 *N*= 35, Not known *N*= 30, **Study 2** Tumour stage, Ta *N*= 8, TIS *N*= 2, T1 *N*= 5, Not known *N*= 11 **Demographics: Study 1 Age**: Mean Age 64.6 (9.50), **Study 2** 69.1(9.40) ** Gender**: **Study 1** male *N*= 85, female *N*= 32 **Study 2** Male *N*= 21 female *N*= 4 **Marital status: Study 1** married *N*= 87, single/never married *N*= 3, Separated/divorced *N*= 17, widowed *N*= 10 **Study 2** married *N*= 19, single/never married *N*= 3 Separated/divorced *N*= 1, widowed *N*= 3 **Smokers:** Not Reported **Employment:** Not reported **Education**: **Study 1** less than high school *N*= 0, High school graduate *N*= 18, Some College *N*= 43 Bachelor’s degree *N*= 32, Post Graduate degree *N*= 24, unknown *N*= 0 **Education**: **Study 2** less than high school *N*= 2, High school graduate *N*= 7, Some College *N*= 8 Bachelor degree *N*= 8, Post Graduate degree *N*= 0, unknown *N*= 1	Convenience	Not reported	Not reported	Mixed methods	1	**Questionnaires:** Demographics: Unspecified questionnaire. Quality of life: European Organisation for the Research and Treatment of Cancer- Quality of Life -Superficial Bladder Cancer-24 (EORTC QLQ-BLS-24). Depression and Anxiety: Brief Symptom Index-18 (BSI-18). Illness Intrusiveness Rating Scale (IIRS)
Krajewski et al., 2017 Poland	To evaluate the pain perception, shift in depression and sexual satisfaction of male patients undergoing cyclic rigid/flexible cystoscopy after TURB for NMIBC	Urology/Urological Oncology Department Wroclaw Medical University	*N*= 100	**Clinical**:—at least one cystoscopy, Surveillance after TURBT for NMIBC **Treatment**- Rigid cystoscopy *N* = 50, Flexible cystoscopy *N* = 50 **Cancer Grade & Stage**: Not reported **Demographics: Age**: 69 + -SD 7.3years (18–86) **Gender**: Male *N* = 100 **Marital status**: Not Reported **Smokers**: Not reported **Employment**: Not Reported **Education:** Not Reported	Convenience		Not Reported	Quantitative	2 Pre and Post cystoscopy	**Questionnaires:** Demographics: Only age reported. Pain: Numeric Rating Scale (NRS). Depression and Anxiety: Hospital Anxiety & Depression Scale (HADS). Sexual Function: The Sexual Satisfaction Questionnaire (Nomejko &Dolifiska-Zygmunt)
Mazur et al., 2023 Poland	To describe the methods of coping with neoplastic disease in men with non-muscle-invasive bladder cancer	Department Urology Wroclaw Medical University	*N*= 100 men	**Clinical**: History of at least 1 TURB **Treatment**-. Number of previous TURB-1- *N* = 33, 2- *N* = 34, ≥ 3-*N*= 31, n/d-*N* = 2.Previous BCG *N* = 17, Previous intravesical Chemotherapy *N*= 5 **Cancer Grade & Stage:** Not reported **Demographics: Age**: 69 (SD 7.3 range 48–86) **Gender**: male-*N*= 100 **Marital status:** married *N*= 60 single *N* = 11, divorced *N* = 5, widowed *N* = 22, n/d- *N* = 2 **Smokers:** Yes *N* = 35, no-*N*= 62, n/d-*N*= 2 **Employment:** Not Reported **Education**: Primary *N* = 34, Secondary/Vocational *N* = 47 Higher-*N*= 17, n/d-*N*= 2	Convenience	Not reported	Not reported	Quantitative	1	**Questionnaires:** Demographics: Unspecified questionnaire. Depression and Anxiety: Hospital Anxiety & Depression Scale (HADS). Coping strategies: miniCOPE questionnaire (Polish adaptation). Sexual function: The Sexual Satisfaction Questionnaire. Pain: Numeric Rating Scale
Miyake et al., 2022 Japan	To investigate the sleep quality of NMIBC patients before, during and after BCG intravesical therapy	Nara Medical University Kashihara, Nara	*N*= 10	**Clinical: Not Reported **Treatment-** Not reported **Cancer stage**: Not Reported **Demographics: Age**: Not Reported **Gender**: Not Reported **Marital status**: Not Reported **Smokers**: Not Reported **Employment:** Not Reported **Education:** Not Reported	Convenience	100%	Not Reported	Quantitative	4 Baseline 4th dose 8th dose 1-month post	**Questionnaires:** Demographics: Unspecified questionnaire. Quality of life: European Organisation for the Research and Treatment of Cancer- Quality of Life (QLQ-C30). Patient Reported Outcomes: International Prostate Symptom Score (IPPS). Functional Assessment of Cancer Therapy Bladder (FACT -BL). Health Survey: Multi-Item Short Form -8 (SF8) Body Composition: standing 8-electrode bioimpedance analysis with InBody 770® Device Real-Time Sleep monitoring: Motion watch 8
Park et al., 2022 Korea	To explore patterns of QOL and differences in social support, self-efficacy, and knowledge levels of depression in NMIBC population	Urological Clinic Tertiary Teaching Hospital Korea	*N*= 278	**Clinical**: Recurrence of disease Nil *N*= 182, 1 *N*= 47, 2 *N*= 20, > 3 *N*= 29 **Treatment**-TURB *N*= 71, TURB + Intravesical therapy *N*= 207 (BCG/MMC) **Cancer Grade & Stage**: Not reported **Demographics: Age**: Mean age 66 (SD 10.6) ** Gender**: Male *N*= 237, Female *N*= 41 **Marital status**: Spouse *N*= 212, No *N*= 66 **Smokers**: Non-smoker *N*= 66, Ex-smoker *N*= 171, Current smoker *N*= 39 **Employment:** Not reported **Education:** Middle school *N*= 56, High school *N*= 105, > College *N*= 117	Convenience	Not Reported	Not reported	Quantitative	1	**Questionnaires:** Demographics: Unspecified questionnaire. Quality of life: European Organisation for the Research and Treatment of Cancer- Quality of Life (QLQ-C30) (Korean version). Research and Treatment of Cancer- Quality of Life -Superficial Bladder Cancer-24 (EORTC QLQ-BLS-24-) (Korean Version). 6 domains used urinary symptoms, malaise, future worries, bloating/flatulence, sexual function, intravesical treatment issues. Depression: Patient Health Questionnaire (PHQ-9). Knowledge of NMIBC: Developed questionnaire. Social support: The Social Supportiveness Scale. Self-Efficacy: Strategies used to by people to promote health (SUPPH). Perceived susceptibility towards and severity of cancer recurrence: Perceived Susceptibility and Severity Scale
Richards et al., 2021 Ireland	To explore the illness perceptions held by patients with NMIBC attending for surveillance cystoscopy or intravesical therapy	Mercy University Hospital Cork	*N*= 118	**Clinical**: **Treatment**- Surveillance cystoscopy *N*= 82, Intravesical therapy *N*= 14. TURBT-*N*= 68, TURBT + Intravesical therapy *N*= 28. Intravesical therapy stage induction *N*= 14, Maintenance *N*= 11 Refractory *N*= 3 **ancer Grade & Stage**: Tumour grade Low *N*= 55, High *N*= 41. CIS *N*= 3, pTa *N*= 75, pT1 *N*= 18, papillary with CIS *N*= 17, **Demographics: Age**: Mean 67.81 (39–85) ** Gender**: Male *N*= 69, Female *N*= 27 **Marital status**: single *N*= 16 Married/life partner *N*= 62, divorced/separated *N*= 8 Widowed *N*= 10 **Smokers**: Non-smoker *N*= 54, smoker *N*= 14, ex-smoker *N*= 28 **Employment:** Employed *N*= 24unemployed *N*= 6, retired/disability *N*= 65, missing *N*= 1 **Education:** Not reported	Convenience	61.5%	Not reported	Quantitative	1	**Questionnaires:** Demographics: Unspecified questionnaire. Brief Illness Perception Questionnaire (B-IPQ). Drawings of their bladder
Smith et al., 2022 USA	To explore the QOL of life of bladder cancer patients with NMIBC, MIBC & metastatic bladder cancer. About financial burden and work disability	Bladder Cancer Advocacy Network Patient Survey Network	Non recurrent NMIBC *N*= 306 Recurrent NMBIC *N*= 272	**Clinical**: Total Non-recurrent NMIBC highest stage diagnosed Non-invasive (NRNMIBC) *N*= 306, Recurrent NMIBC (RCNMIBC) *N*= 272 Time to recurrence: 1–5 years **Treatment**- not reported **Cancer Grade & Stage**: First stage of bladder cancer diagnosed. NRNMIBC Non-invasive *N*= 304, invasive *N*= 1 don’t know *N*= 1. RCNMIBC *N*= 270**,** invasive cancer *N*= 2 **Demographics: Age**: NRNMIBC-68.3(9.0) (69), RCNMIBC- 66.7 (8.4) (68) **Gender**: NRNMIBC-male *N*= 166, female-*N*= 93, missing *N*= 47, RCNMIBC- male *N*= 127, female *N*= 112, missing *N*= 33 **Marital status**: NRNMIBC-married *N*= 188, living with partner *N*= 7, divorced *N*= 26, widowed *N*= 17, separated *N*= 3single never married *N*= 16, missing *N*= 49. RCNMIBC married *N*= 185, living with partner *N*= 6, divorced *N*= 23, widowed *N*= 15, separated *N*= 0, single never married *N*= 5, missing *N*= 38 **Smokers**: Not reported **Employment:** Not reported **Education:** NRNMIBC- some high school *N*= 1, high school graduate *N*= 16, Some college (did not complete) *N*= 61, College graduate *N*= 86, Post-college Graduate *N*= 95, missing *N*= 47. RCNMIBC- some high school *N*= 0, high school graduate *N*= 16, Some college (did not complete) *N*= 63, College graduate *N*= 67, Post-college Graduate *N*= 92, missing *N*= 34	Convenience	Not reported	Not reported	Quantitative	1	**Questionnaires:** Demographics: Unspecified questionnaire. Quality of life: European Organisation for the Research and Treatment of Cancer- Quality of Life (QLQ-C30). Bladder Cancer Index (BCI). Financial Toxicity: Comprehensive Score for Financial Toxicity (COST). Work productivity: Work Productivity and Activity Impairment Questionnaire: General health (WPAI:GH)
Tan et al., 2020 United Kingdom	To explore the patient experience and perception of being diagnosed with bladder cancer and the effect on their health-related quality of life	52 UK hospitals	*N*= 213 *N*= 20 Qualitative	**Clinical**: Participants completed Brief IPQ and Interviews > 6 months after the new diagnosis. New tumour *N*= 135, Recurrence *N* = 78, **Treatment**-Previous cystoscopies < 2 *N* = 66, 2–5 *N* = 92, > 6 *N* = 47 not known *N* = 8 **Cancer Grade & Stage**: Tumour grade: G1 *N* = 36, G2 *N* = 99, G3 *N*= 71 Not known *N* = 7. CIS *N* = 3, pTa *N* = 156, pT1 *N* = 47, Not known *N* = 7 Disease Risk: Low *N* = 18, Intermediate *N*= 105, High *N* = 83 Not Known *N* = 7 **Demographics: Age**: 74 Years (67.1–81.1) **Gender**: Male *N* = 170, Female *N*= 43 **Marital status**: Not Reported **Smokers**: Non-smokers *N* = 56, Ex-smoker *N* = 129, Current smoker *N* = 18, Not known *N* = 10 **Employment**: Fulltime/part-time/homemaker/voluntary *N* = 45, Retired *N* = 161, Disability/unemployed *N* = 4, Missing *N* = 3 **Education:** No formal education *N* = 8, High school *N* = 56, GCSE *N* = 39, A-Levels *N* = 20, University /Higher degree *N* = 31, Not known *N* = 59	Convenience	57%	Not reported	Mixed methods	1	**Questionnaires:** Demographics: Unspecified questionnaire. Cognitive illness: The brief Illness Perception Questionnaire (Brief IPQ) Semi-structured telephone interviews- explore patients
Van Der AA et al., 2009 Netherlands	To explore the sexual function of patients recently diagnosed with primary or recurrent NMIBC	7 Hospitals in Netherlands (Multicentre)	*N*= 150	**Clinical:** Sexually inactive **(SI):** *N*= 55 Sexually Active **(SA):** *N*= 87 **Treatment**-Not recorded **Cancer Grade & Stage**: pTa, pT1, Grade1 or 2 **Demographics**: **Age:** Median range **(SI)** 72 (35–89) **(SA)** 66 (37–83) **Gender:** Male *N*= 105, Female *N*= 37. Male **(SI)** *N*= 35-(33%) Female *N* = 20 -(54%). **(SA)** Male *N*= 70 (67%), Female *N*= 17-(46%) **Marital status: (SI)** married/cohabitation *N*= 37-(33%), single *N* = 4, divorced *N*= 5, widow/er **(SA)** married/cohabitation *N*= 75, single *N*= 1, divorced *N*= 7, widow/er *N*= 2 **Smokers: (SI)** YES *N*= 45, No-*N*= 9 **(SA)-***N*= 75, No *N* = 10 **Employment: (SI)**- Steady Job *N* = 10, Voluntary Job-*N*= 6, Disablement Insurance, *N*= 7, Retirement-*N*= 32** (SA)**-Steady Job- *N* = 30, Voluntary Job-*N*= 7, Disablement Insurance, *N*= 2, Retirement-*N*= 48 **Education**: **(SI)** Vocational-*N*= 14, Secondary school-*N*= 17, Advanced Secondary School-*N*= 7, Higher Vocational *N* = 10, University-*N*= 6, **(SA)** Vocational-*N*= 17, Secondary school-*N*= 27, Advanced Secondary School-*N*= 10, Higher Vocational *N*= 24, University-*N*= 9	Convenience	*N*= 142 95%	Not reported	Quantitative	2 Start of surveillance < 3 months after the diagnosis of primary or recurrent	**Questionnaires:** Demographics: Unspecified questionnaire. Quality of life: General state of health- assessed with Visual Analogue Scale (VAS) Sexual health: validated subset 8 questions on sexual performance from Research and Treatment of Cancer- Quality of Life -Superficial Bladder Cancer-24 (EORTC QLQ-BLS-24-)
Vaioulis et al., 2020 Greece	To evaluate the quality of life and anxiety response in patients having TURB and intravesical therapy		*N*= 117 9 withdrew for progression *N*= 108	**Clinical**: **Treatment**- Patients had TURB and either Epirubicin *N*= 17 or BCG intravesical therapy *N*= 91 **Cancer Grade& Stage**: Ta -T1 Low grade *N* = 17, T1 high grade *N*= 91 **Demographics: Age**: < 66 years *N*= 40, 66 + *N*= 68 **Gender**: male *N* = 97, Female *N* = 11 **Marital status**: unmarried *N* = 1, married *N*= 92, Divorced *N*= 8, widowed *N* = 4, cohabitation *N*= 2 **Smokers**: No *N* = 12 Ex-smokers *N* = 60, yes *N* = 36 **Employment:** Employed *N* = 20, Retired *N*= 83, Unemployed *N* = 4, Household *N* = 1 **Education:** No primary Education *N* = 3, Primary Education *N*= 49, Secondary education *N*= 26, Higher, Education *N*= 26, Masters *N* = 4	Convenience	Not reported	Not reporter	Quantitative	4 2 weeks preoperatively 3,6,12 months postoperatively 3,6,12 months postoperatively	**Questionnaires:** Demographics: Unspecified questionnaire. Quality of life: The 36-Item Short Form Health Survey (SF-36). Anxiety: The State-Trait Anxiety Inventory questionnaire (STAI-YI)
Wei et al., 2014 China	To evaluate the quality of life and local symptoms of patients with NMIBC receiving intravesical treatments	The Peoples Hospital of Guangxi Zhuang Autonomous Region	*N*= 106	**Clinical**: **Treatment**-Pirarubcin (intravesical) 40mg weekly for 6 weeks then monthly for 12 months **Cancer Grade & Stage**: Intermediate risk -*N*= 71, High Risk *N*= 35 **Demographics: Age:** 61 +—15.5 years. < 40–12, 40–60 *N*= 34, > 60 *N*= 60 **Gender**: Male *N*= 82, Female *N*= 24 **Marital status**: Not Reported **Smokers**: Not Reported **Employment**: Not Reported **Education:** Not reported	Convenience	Not reported	Not reported	Quantitative	2 Pre instillation and Post instillation	**Questionnaires:** Demographics: Unspecified questionnaire. Quality of life: European Organisation for the Research and Treatment of Cancer- Quality of Life (QLQ-C30.Chinese Version). Local symptoms: Core Lower Urinary Tract Symptom Score (CLSS)
Wildeman et al., 2021 Netherlands	To explore which psychosocial issues patients are confronted with during intravesical treatment with BCG, MMC and the impact on daily life, social, emotional, and physical well-being	Urological Outpatients Franciscus Gastthuis Hospital Rotterdam	*N*= 80	**Clinical**: **Treatment-** BCG-*N*= 62, MMC *N*= 18 **Cancer Grade & Stage**: Not reported **Demographics: Age**: 40–94 Years Median age-69.7 **Gender**: Male *N*= 66, Female *N*= 14 **Marital status**: partner *N*= 63 **Smokers**: Not reported **Employment**: *N*= 17 **Education:** Not reported	Convenience	Not reported	*N*= 16 Due to the progression of BC *N*= 6 withdrew from treatment	Quantitative	Induction (week 6), 6 months (2) BCG-maintenance in years 1, 2, 3 (1)	**Questionnaires:** Demographics: Unspecified questionnaire. Quality of Life: European Organisation for the Research and Treatment of Cancer- Quality of Life -Superficial Bladder Cancer-24 (EORTC QLQ-BLS-24-) (Dutch Version). Psychosocial: Psychosocial distress screening tool (Dutch Version), Distress thermometer (DT). Question to evaluate satisfaction with a urological oncology nurse
Zhang et al., 2020 China	To evaluate the characteristics of depression, anxiety and illness perception in NMIBC patients and explore the value of illness perception in predicting depression and anxiety		*N*= 101	**Clinical**: **Treatment-** Completed initial TURB +—adjuvant maintenance intravesical therapy **Cancer Grade & Stage**: 0 *N*= 81 1 *N*= 20. Grade 0 *N*= 31, Grade 1 *N*= 48, Grade 2 *N*= 22. Single lesion Yes *N*= 75, No *N*= 26 **Demographics: Age**: 63.9 +—13.8 **Gender**: Male *N*= 74, Female *N*= 27 **Marital status**: Married *N*= 87 Divorced/Widow *N*= 14 **Smokers**: Yes, *N*= 38. No *N*= 63 **Employment**: Employed *N*= 24, unemployed *N*= 77 **Education:** Primary education *N*= 47, High school *N*= 32, Higher education *N*= 22	Convenience	Not reported	Not reported	Quantitative	3 Baseline,3 & 12 months	**Questionnaires:** Demographics: Unspecified questionnaire. Illness perception: Brief Illness Perception Questionnaire (B-IPQ) Chinese version. Anxiety and Depression: Hospital Anxiety & Depression Scale (HADS

*HADS*, hospital depression and anxiety scale; *BC*, bladder cancer; *BCG*, Bacillus Calmette-Guerin; *MMC*, Mitomycin C; *SI*, sexually inactive; *SA*, sexually active; *TURBT*, transurethral resection of bladder tumour; *RT*, radical radiotherapy; *RC*, radical cystectomy; *n/d*, no data; *EORTC QLQ-BLS-24*, European Organisation for the Research and Treatment of Cancer- Quality of Life -Superficial Bladder Cancer-24; *N/A*, not applicable; *Brief IPQ*, Brief Illness Perception Questionnaire; *CIS*, carcinoma in situ; *GCSE*, General Certificate of Secondary Education; *QOL*, Quality of Life **-Authors contacted for demographics – no response

Results of methodological quality assessment are presented in Table 2.

**Table 2:**
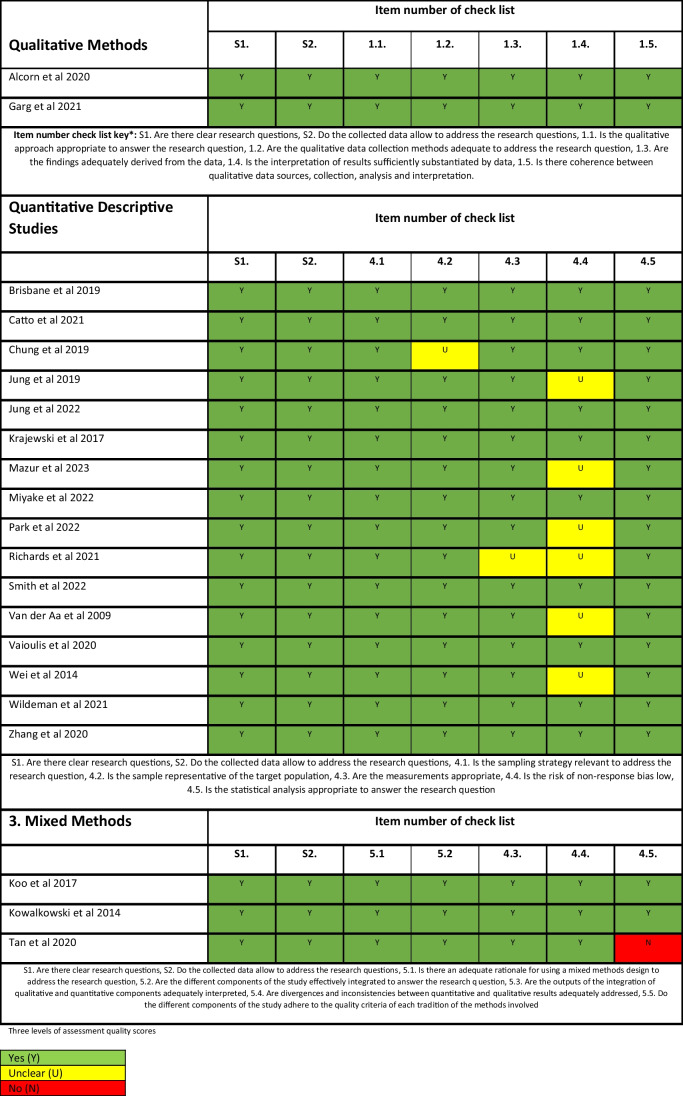
Quality appraisal of included studies

**Fig. 2:**
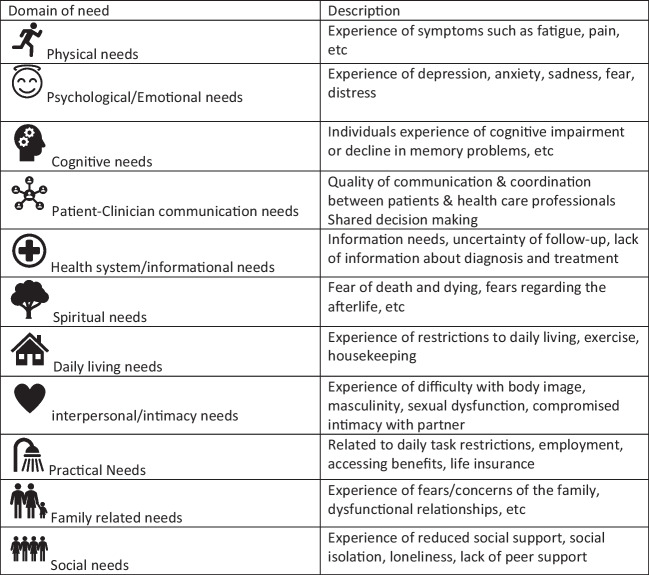
Supportive care domains

The studies included qualitative studies (*n* = 2), quantitative (*n* = 16) and mixed methods (*n* = 3). Overall, the methodological quality of the studies was credible, with only one study (Tan et al., 2020) that did not meet all the quality assessment criteria (Fig. 2).

### Frequency of identified supportive care needs

The supportive care needs of the participants included in this review were classified according to the eleven domains (see Table 3). The supportive care needs comprised of the following in order of significance: psychological/emotional, *n* = 16/21: 76%, physical *n* = 16/21:76%, practical *n* = 8/21:38%, interpersonal/intimacy *n* = 7/21:33% and health system/informational *n* = 5/2:23%, social *n* = 4/21:19%, patient/clinician communication *n* = 3/21:14%, spiritual *n* = 2/21:9.5%, and daily living *n* = 1/21:5%. Participants enduring intravesical therapy had intimacy concerns [27, 45, 49] and fear of contaminating their partners [47, 51]. They also requested information and support to access sexual well-being interventions [27, 45, 47, 51].

**Table 3:** Frequency of supportive care need by domain

Study	Physical Needs	Psychological/ Emotional Needs	Cognitive Needs	Patient-Clinician communication	Health System/ Information Needs	Spiritual Needs	Daily Living Needs	Interpersonal/ Intimacy Needs	Practical Needs	Family Related Needs	Social needs	Number of domains explored within each study
Alcorn et al. 2020	**✓**	**✓**	**-**	**-**	**✓**	**✓**	**-**	**-**	**-**	**-**	**✓**	**5**
Brisbane et al. 2018	**✓**	**-**	**-**	**✓**	**-**	**-**	**-**	**-**	**-**	**-**	**-**	**2**
Catto et al. 2021	**✓**	**✓**	**-**	**-**	**-**	**-**	**-**	**✓**	**✓**	**-**	**-**	**4**
Chung et al. 2019	**-**	**✓**	**-**	**-**	**✓**	**-**	**-**	**✓**	**✓**	**-**	**-**	**4**
Garg et al. 2018	**✓**	**✓**		**✓**	**✓**	**-**	**-**	**-**	**✓**	**✓**	**-**	**6**
Jung et al. 2020	**✓**	**-**	**✓**	**-**	**-**	**-**	**-**	**-**	**-**	**-**	**-**	**2**
Jung et al. 2022	**✓**	**✓**	**✓**	**-**	**-**	**-**	**-**	**-**	**✓**	**-**	**-**	**4**
Koo et al. 2018	**✓**	**✓**	**-**	**-**	**-**	**-**	**✓**	**-**	**✓**	**✓**	**✓**	**6**
Kowalkowski et al. 2014	**-**	**✓**	**-**	**-**	**-**	**-**	**-**	**✓**	**-**	**✓**	**-**	**3**
Krajewski et al. 2017	**✓**	**✓**	**-**	**-**	**-**	**-**	**-**	**✓**	**-**	**-**	**-**	**2**
Mazur et al. 2022	**✓**	**✓**	**-**	**-**	**-**	**✓**	**-**	**-**	**-**	**✓**	**-**	**4**
Miyake et al. 2022	**✓**	**-**	**-**	**-**	**-**	**-**	**-**	**-**	**-**	**-**	**-**	**1**
Park et al. 2021	**✓**	**✓**	**-**	**✓**	**-**	**-**	**-**	**-**	**-**	**✓**	**-**	**4**
Richards et al. 2021	**✓**	**✓**	**-**	**-**	**✓**	**-**	**-**	**-**	**-**	**-**	**-**	**3**
Smith et al. 2022	**✓**	**-**	**-**	**-**	**-**	**-**	**-**	**✓**	**✓**	**-**	**-**	**3**
Tan et al. 2020	**-**	**✓**	**-**	**-**	**✓**	**-**	**-**	**-**	**-**	**✓**	**-**	**3**
Vaiouslis et al. 2021	**✓**	**✓**	**-**	**-**	**-**	**-**	**-**	**-**	**-**	**-**	**-**	**2**
Van de Aa et al. 2009	**-**	**✓**	**-**	**-**	**-**	**-**	**-**	**✓**	**-**	**-**	**-**	**2**
Wei et al. 2014	**✓**	**-**	**-**	**-**	**-**	**-**	**-**	**-**	**✓**	**-**	**✓**	**3**
Wildeman et al. 2020	**✓**	**✓**	**-**	**-**	**-**	**-**	**-**	**✓**	**✓**	**✓**	**✓**	**7**
Zhang et al. 2020	**-**	**✓**	**-**	**-**	**-**	**-**	**-**	**-**	**-**	**-**	**-**	**1**
**Number of domains explored across all studies**	**16**	**16**	**2**	**3**	**5**	**2**	**1**	**7**	**8**	**7**	**4**	

### Supportive care needs

#### Psychological/emotional needs

Psychological and emotional needs were prominent throughout this literature review, with sixteen out of twenty-one studies identifying them as prominent needs [27–29, 36, 38, 40, 41, 43, 44, 46, 47, 49–53]. Psychological and emotional symptoms were prevalent from the initial diagnosis and throughout their treatment and survivorship phases [28, 36, 37, 51, 52]. The participants who had either transurethral resection of bladder tumour and intravesical therapy reported anxiety, depression [28, 38] and uncertainty [15, 40]. Uncertainty was identified to be related to their initial diagnosis, treatment regime and fear of cancer recurrence [26, 38, 41, 43, 52, 54]. People with NMIBC were reported to have a unique burden due to the high recurrence rates and frequent invasive surveillance regimes, which elevated distress due to cancer-related uncertainty [5, 28, 40]. Many participants reported needing assistance making life decisions, such as treatment decisions and the potential support they may require during their treatment phase. These decisions were in the context of uncertainty, which impacted their psychological and emotional well-being [15].

In one study, Jung et al. (2022) reported that NMIBC individuals met at least one criterion for post-traumatic stress disorder. Post-traumatic stress disorder is defined as a harmful or life-threatening event that can impact an individual’s emotional, physical, social or spiritual well-being [40].

Post-traumatic stress symptoms (PTSS), particularly uncertainty, directly impacted NMIBC participants’ quality of life. Jung et al. (2022) reported that uncertainty in the NMIBC population is related to fear of recurrence and the long-term effects of treatments. The more uncertain patients felt, the greater their quality of life declined. Individuals with NMIBC with a higher uncertainty rate were identified predominately as younger male participants, unaware of their disease status, with lower social support and income [40].

Participants who received intravesical therapy reported a significant impact on their quality of life, experiencing greater depression and difficulty with emotional coping and physical well-being [38, 40, 52]. They also stated concerns about transmitting disease to their partners, especially when being intimate [51]. Many participants experienced shock, worry and anxiety with their initial diagnosis of bladder cancer [52].

“Yes. My emotional it affected my emotional well-being principally worry and anxiety” pg. 674 [52].

NMIBC participants reported using various coping strategies for their stress, including active coping, acceptance of their condition and using a sense of humour to cope with specific situations. Unhelpful strategies included denial, avoidance of the situation and substance abuse [29].

#### Physical needs

Physical symptoms were prominent across the majority of studies. Participants reported their main concerns were urinary symptoms [26, 42, 45, 48, 49], pain [38, 41, 48, 49, 54] sleep difficulties [28, 42, 45, 49] and fatigue [49, 55]. Individuals receiving intravesical therapy experienced fluctuating symptoms that were more significant whilst on treatment. Some participants expressed the impact of their urinary symptoms as painful; for some, they reported to have lasted 6–7 h, which impacted their quality of life [36]. Poor sleep quality was due to urinary symptoms such as nocturia, frequency, urgency and urinary incontinence [26, 28, 41, 42, 44, 54]. Urinary symptoms also affected participants’ ability to socialise with family and friends as they had to plan their activities around a bathroom location, sometimes opting not to engage in social outings [36].

Pain was commonly identified as a physical symptom for NMIBC participants [28, 38, 41, 42, 45, 49]. Individuals reported experiencing pain whilst enduring cystoscopy procedures for their surveillance protocol. Krajewski et al. (2017) reported that participants experienced more pain with a rigid cystoscopy than with a flexible cystoscopy. Participant pain was attributed to an association with recalled pain from previous cystoscopy experiences, which was increased slightly by the participants’ anxiety and anticipatory fear [41]. Some participants enduring intravesical therapy treatment experienced more significant pain post-treatment, which they did not expect. Participants commented that the patient information brochure did not detail this side effect [36, 48, 49].

“There are really painful downsides, and maybe the difference is that the literature says that there are downsides, but they don't say it can be quite traumatic …. This is one of the big problems it was that 6–7 h of intense pain …It was up at the higher level of pain than there was in the literature” pg. 109 [36].

#### Practical needs

Financial support was the greatest identified practical need within this review. The participants were representatives from various countries, including the UK, Canada, China, and the USA; despite these countries being developed, participants reported financial burden and the loss of work hours whilst having treatment for NMIBC [15, 38, 40, 45, 48]. Some NMIBC people experienced financial toxicity, which impacted their ability to work, mainly due to multiple outpatient visits for their treatment regimes. One study by Chung et al.(2019) (*n* = 586) reported that 66% of participants wanted assistance to access financial information [15]. Wei et al. (2014) reported that financial difficulties were present among participants commencing intravesical therapy prior to treatment and were significantly higher following treatment. This was attributed to increased urinary symptoms, discomfort and loss of productive work hours [48].

Catto et al. (2021) identified that NMIBC people less than 65 years old having transurethral resection of bladder tumours suffered from financial toxicity due to their treatment regime and inability to attend to their work. One study by Jung et al. (2022) reported that lower income was associated with a lower quality of life and higher uncertainty, leading to increased stress levels for the NMIBC participants [40]. In contrast, Koo et al.(2017) found that 73% of participants felt more capable of meeting work and home responsibilities following their treatment as they were aware of their treatment plan and did not have anxiety prior to treatment [28].

#### Interpersonal/intimacy needs

Non-muscle invasive bladder cancer people expressed a desire for assistance with sexual and intimacy needs [15, 38, 41, 45, 47, 49, 51]. One issue identified was the fear of contaminating their partner during sexual intercourse [37, 38, 47, 51]. Other issues included participants requiring assistance with relationships and strategies to assist with their partners’ understanding of their cancer [15, 38, 51]. A decline in sexual function and enjoyment was experienced by participants who were increasing in age and had other health conditions. Several participants experienced sexual dysfunction before diagnosis [38, 47, 51]. Sexual issues were identified as a concern in participants receiving intravesical therapy. In one cohort, 50% of participants aged 40–50,33%, 50–59, and 19% aged 70–79 experienced sexual issues that impacted their intimate relationships [49]. Women were less likely to be sexually active (56%) than men (31%), and those women who were sexually active experienced vaginal dryness [51]. In comparison, men experienced erectile and ejaculatory dysfunction [47, 51]. Participants on current intravesical treatment stated that it impacted their relationships due to their perceived loss of intimacy [51]. Some participants found sharing their sexual concerns with their partners was beneficial. Effective communication between the couple provided an opportunity to re-establish a sexual relationship following a diagnosis of NMIBC. At the same time, others reported difficulty initiating the conversation and sought professional assistance [51].

“Well, obviously, for sex, it's different. As far as the marriage goes, it really made it stronger. Like I said, she was there for me the whole time. And I think we bonded a little closer even. We've been married for [over two decades], so it's, I mean, we were pretty close before that. And obviously [bladder cancer] changed our sex life a little bit. We still have sex, but it's a little different now” pg.148 [51]

#### Family and related needs

Participants expressed the importance of family and support with their diagnosis and treatment [28, 37, 49, 51, 52, 54]. Those with family support reported higher quality of life scores [43]. Partners of people with NMIBC were identified as the primary support for their loved ones, offering practical support with managing appointments and helping with lifestyle changes, such as quitting smoking and encouraging physical exercise [37].

Some NMIBC participants waiting for treatment felt they were taking issues out on their support people, which intensified their feelings of guilt and anxiety. However, 82% felt improvement in their relationship with family and friends following their cystoscopy procedure [28]. There was an association between higher quality of life scores for people with NMIBC and those who reported having a supportive partner or family member. The benefit of their support is that people provide effective communication, assistance and support [43, 51, 52].

“I had a belief that I wouldn't succumb as in, you know, it wouldn't be fatal for me, but then I had to that kind of positive thing. I had positive thinking. I didn't really tell my kids too much. My sister is pretty sympathetic, my sister was pretty helpful” pg. 674 [52].

#### Health system and informational needs

Several studies reported a lack of information for participants [27, 28, 36, 44, 52, 53] and included sub-optimal support on managing physical symptoms and navigating the healthcare system [15, 36, 37, 44, 52]. Due to the continual surveillance protocols for people with NMIBC, timely information provided by healthcare professionals was paramount. Some patients experienced shock with their initial diagnosis having assumed it to be either a urinary tract infection or a prostate problem [52]. Other participants had seen television advertisements encouraging them to have their symptoms reviewed by a doctor [52]. One study by Richards et al. (2021) indicated that patients’ knowledge about smoking and its causal factor to NMIBC was poor [44].

Several participants felt the surveillance cystoscopy process could be improved by providing time to discuss findings and other lifestyle changes immediately following their procedure [37]. Some participants experienced anxiety and stress at the delay in their procedure [37] or the time in receiving their pathology results [28]. In contrast, patients who had subsequent follow-up consultations felt they understood the process and were less stressed. Understanding the processes and providing adequate time for discussion made the participants feel they were actively involved in their treatment process [52].

#### Social needs

Four studies [28, 36, 48, 49] acknowledged the impact of NMIBC treatment on participants’ social needs. Participants reported mutual feelings, including withdrawing from those close to them, which caused feelings of isolation [28]. Social interactions had decreased following post-intravesical therapy, often due to urinary symptoms and the requirement to be close to a bathroom [28, 36, 49]. Some participants described feeling isolated but preferred to stay at home as they did not want anyone to see them suffering from discomfort from their urinary symptoms [36].

“I had to be on the toilet or next to the toilet wearing incontinence pads because I couldn’t do anything… it has prevented me going out for a drink… knew every toilet on the (name of town) seafront. It’s pre-planning” pg. 109 [36]

#### Patient and clinician communication needs

Participants experienced both positive and negative interactions with healthcare professionals [28, 36, 37, 51, 52]. The interactions involved process factors such as the timing of their surveillance. Some participants wanted more control over their treatment regimes, particularly regarding the necessity and frequency of their surveillance cystoscopies [28]. They reported that they would like to be involved in making their treatment decisions regarding when they will have their follow-up cystoscopy; other participants were happy to leave this to the urologist [28].

Some participants experienced negative interactions with healthcare professionals, particularly after their surveillance cystoscopy. They would have liked a discussion and explanation of the findings immediately after their procedure, which did not always happen [37]. One participant was informed of her cancer diagnosis via email from another surgeon [37]. Some participants who received intravesical treatment during the BCG shortage were not given explanations of why they were receiving a different medication or a reduced amount of BCG. The lack of information about this change in their treatment regime resulted in feelings of anxiety and worry for NMIBC people [37].

The NMIBC participants experienced positive interactions with their healthcare professionals and had an improvement in their quality of life scores [43]. They were more likely to discuss and receive treatment for their urinary symptoms due to feeling comfortable with their healthcare professional [26]. Some individuals received an out-of-hours phone call from the treating physician and found reassurance in receiving this call [37]. Several participants expressed that they preferred continuity of care, having the same urologist or health care professional perform their surveillance cystoscopy. When this did not occur, it caused some participants increased anxiety. In contrast, others were comforted by having a “different set of eyes” to view their bladder pg.126 [28].

One study [53] suggested that having access to a healthcare professional was fundamental to rural patients when urologists and healthcare professionals are limited. Some participants described feelings of anxiety and concern with the lack of urologists, and others felt frustrated with the delay in waiting for their procedures [53]. Several participants appreciated having the telephone number of a nurse navigator to contact if they needed assistance or advice. Ensuring that patients had access to the point of care prevented hospital admissions, provided patient satisfaction and improved patient experiences for NMIBC participants [37].

#### Cognitive needs

Cognitive needs were identified in two studies [39, 40]. It was measured using the PROMIS (Patient-Reported Outcomes Measurement Information System Applied Cognition-Abilities short form and PROMIS Applied General Concerns form. Cognition abilities refer to an individual’s capacity to plan, reason and understand complex ideas. It is often associated with positive connotations [56]. For participants with general cognition concerns, it is often associated with symptoms from their disease or treatment and can negatively affect the individual. The mean score ranges from 8 to 40, with the higher score indicating better cognitive function. For the participants included in this study (*n* = 376), the mean score (SD) was 31.9 (7.5) (SD) for cognition abilities [40]. NMIBC participants in this study reported a mean score of 14.4 (7.5) (SD) for general cognition concerns. Females affected by NMIBC reported higher levels of positive self-assessment of their cognitive functioning abilities, and this was associated with lower post-traumatic stress disorder and higher perceived quality of life scores. Participants who were currently having treatment had multiple comorbidities and more cognitive concerns and experienced a lower quality of life [40].

#### Spiritual needs

Only two studies identified spiritual needs in this review [36, 54]. Some participants identified spiritual needs as an excellent support, mainly assisting with their coping methods [36, 54]. Older participants with lower education levels were more likely to use religion as a coping strategy. Participants who experienced depressive symptoms were also weakly associated with using religion to cope [54]. Some participants gained strength from their church community, which made them feel psychologically and emotionally stronger to cope with their treatment [36].

#### Daily living needs

Across the studies, only one study, Koo et al. (2017), identified daily living needs as a concern. People affected by NMIBC expressed having difficulty performing daily activities such as housework or cooking before their cystoscopy procedure. Participants attributed the impact of stress, anxiety and feelings of apprehension prior to their treatment as the cause. These feelings were resolved following their treatment, and participants felt they could meet their home and work responsibilities [28].

## Discussion

This integrative systematic review set out to identify the supportive care needs of people diagnosed with NMIBC and to report the most frequently reported needs in the literature. Identifying the supportive care needs will assist in guiding future interventions for service delivery. Supported care needs are defined as the individual’s request for general support or an identified problem prioritised by the individual when diagnosed or treated for cancer [19, 57]. Unmet supportive care needs refer to absence of or assistance in support of an identified problem of the NMIBC individuals. Unmet needs can occur from diagnosis throughout treatment and into survivorship or palliative phases [21]. NMIBC participants experienced a unique burden due to the numerous surveillance procedures required, high recurrence rate, and invasive treatments, such as cystoscopy and intravesical treatments. This systematic review identified psychological/emotional and physical domains as the foremost supportive care needs reported across the 21 studies. The NMIBC participants who were newly diagnosed or receiving intravesical therapy experienced more significant unmet needs, particularly with psychological (worry, anxiety, uncertainty and fear of cancer recurrence) and physical symptoms (pain urinary issues, and fear of contaminating their partners with intimacy).

Psychological and emotional needs have been identified as prominent in other reviews on genitourinary cancers [4, 18, 25, 35, 58, 59]. NMIBC participants experienced depression, anxiety and cancer-related uncertainty from diagnosis, throughout their treatment phase, and into survivorship. The feeling of uncertainty in illness pertains to the cognitive state or inability to determine or categorise an event or outcome that cannot be predicted accurately [60]. Cancer-related uncertainty affects the psychosocial adaptation and the effects of the disease on individuals [61]. The NMIBC participants reported enduring worry about cancer recurrence [26, 38, 41, 43, 52, 54]. There is a known relationship between uncertainty, emotional distress, and diminished quality of life which can produce increased anxiety and depression comparable to post-traumatic stress symptoms [39]. The emotional and psychological elements interrelate with other supportive care domains, providing a biopsychosocial model of care [22]. The biopsychosocial model encompasses physical, cognitive, spiritual, intimacy, family, and social needs among the NMIBC population. Healthcare professionals are uniquely positioned to promote screening and assessment of NMIBC people, using patient-reported outcomes measures (PROMS) that capture the physical, emotional/psychological, cognitive, patient-clinician interactions, health system/informational, spiritual, daily living, interpersonal, intimacy, practical and social domains of the needs individuals throughout their cancer continuum [4, 18, 62]. Routine screening for supportive care needs in clinical practice will identify and timely address NMIBC individuals’ unmet needs. Identifying the supportive care needs of this population provides a holistic approach to improving physical and mental well-being and quality of life among NMIBC participants [63].

Physical needs were primarily represented within the NMIBC population, with many participants reporting urinary symptoms as most bothersome [26, 42, 44, 45, 48]. Other symptoms reported included fatigue [49], pain [38, 41, 45, 48, 49, 54] and sleep deprivation [28, 49]. Preparing NMIBC individuals for the potential side effects of their treatment is paramount. Pre-treatment education should be provided using various learning styles; written, verbal, group, individual, and online formats; varying learning styles will promote knowledge and education to prepare NMIBC participants to shed light on uncertainties and assist in minimising their anxiety. Furthermore, it will promote identifying potential side effects of their treatment and timely interventions to assist with self-management [28, 52].

Financial toxicity was the greatest identified practical need within this integrative review. Financial toxicity refers to the financial burden and distress that can occur for patients and their family members as a result of their cancer treatment. It can impact all aspects of their cancer care, from imaging to medical therapy and long-term side effect management [64]. Financial difficulties were attributed to the long-term follow-up and frequent surveillance cystoscopies, which led to participants requiring time off work. Healthcare professionals must consider financial circumstances when developing future financial support interventions for NMIBC people. The impact of the increased cost of living worldwide due to the effects of the global COVID-19 pandemic, climate change and the war in Ukraine will continue to affect health care [65]. Reducing work hours and the rise in the cost of living will present a challenge for NMIBC patients in the future. Lower-income status has been identified as contributing to decreased quality of life and higher uncertainty, leading to increased patient stress levels [5]. Healthcare professionals must consider financial implications for NMIBC individuals when developing future supportive care interventions. It is paramount that NMIBC people are screened early in their care pathway with relevant patient-reported outcome measures to identify their risk of financial toxicity. Identifying individuals at risk will provide early intervention into the patient’s journey.

Health system, information and patient-clinician communication needs were reported to be less bothersome to NMIBC people in this systematic review. Other systematic reviews reporting on genitourinary cancers noted that health systems information and patient and clinician communications were considered a greater participant need [4, 18, 25, 31, 35, 58, 59]. Despite fewer NMIBC participants not reporting information and communication as a significant unmet need, it was still a concern for some individuals. Several participants experienced difficulty initiating sexual intimacy conversations with healthcare professionals due to embarrassment and a lack of support in accessing the interventions. Similar findings in other cancer populations have been well-documented in the literature [4, 18, 31, 35, 66]. A systematic review by Bessa et al. (2020) investigated the sexual health needs of bladder cancer patients. It revealed a paucity of studies, including the NMIBC population. Therefore, this current review provides new insights into the intimacy and interpersonal needs of the NMIBC population. Participants expressed a need for information on relationship strategies [27], initiating conversations with their partners and healthcare professionals [51] and guidance from healthcare professionals to assist in accessing interventions [27]. Some participants described positive interactions with clinician communication, such as being included in treatment decisions and receiving after-hours phone follow-ups [28, 37]. Other participants stated they wanted more information and communication regarding changes to their treatment regime [53]. Partners were identified as the primary support for NMIBC patients. Participants with family, peer and healthcare provider support reported improved quality of life [37, 43] and enhanced coping strategies [54].

Rural patients expressed that the lack of urologists and healthcare professionals in their community caused anxiety and concern. Participants appreciated the contact details of the nurse navigator. Nurse navigators provided patients satisfaction and timely response to patients’ concerns. Telephone consultations delivered by nurses have been utilised in the follow-up care of oncology patients since the late 1990s. A literature review by Cox et al. (2003) showed it to be acceptable, effective and appropriate for elderly and geographically isolated people [67]. It has become an essential element of clinical practice since the COVID-19 pandemic [68].

## Limitations

Although this systematic review followed a registered priori protocol and a structured and rigorous process based upon the PRISMA guidelines to promote reproducibility, limitations were noted [32]. Most of the studies were cross-sectional, representing a snapshot in time and did not consider changes in supportive care needs over time. One of the challenges of this review was the heterogeneous methodologies, and our findings are constrained due to the methodological limitations of the studies included. This review only included articles in the English language and may have limited the applicability of our findings to other populations. One of the challenges of this review is that the NMIBC population includes patients with low-risk disease who require cystoscopy surveillance only, whilst other participants had high-risk NMIBC. The treatment approach will be more intensive with intravesical therapy for high-risk NMIBC people, and their supportive care needs may differ. However, this integrative review has facilitated a summation of the evidence for the supportive care needs of NMIBC, which have been absent in the current literature.

## Clinical Implications and Conclusion

Non-muscle invasive bladder cancer is unique as it makes up the majority of bladder cancer diagnoses, requires lifetime surveillance, has a high recurrence rate and has uncertainty of prognosis. Previous studies have yet to identify the supportive care needs of the NMIBC population. This integrative review has highlighted the critical unmet needs of NMIBC participants. In particular, it has revealed that emotional, psychological and physical needs are currently not met. Nurses are at the forefront of the NMIBC participant’s healthcare journey and use patient-reported outcomes measures to identify their supportive care needs. Identifying participants’ supportive care needs requires regular screening, assessment, and timely intervention.

Future research should include regular assessment to review NMIBC individual’s supportive care needs (PROMS) throughout their cancer continuum, as supportive care needs are dynamic and may vary over time. Identifying the supportive care needs will contribute to developing future interventions to improve patients’ experiences living with a non-muscle invasive bladder cancer diagnosis.

## Supplementary Information

Below is the link to the electronic supplementary material.

Supplementary file1 (DOCX 16 KB)

Supplementary file2 (DOCX 26 KB)

## Data Availability

The data to support the findings of this study are available in the link titled supplementary information.
