# Supplementary material for: Identifying the supportive care needs of people affected by non-muscle invasive bladder cancer: An integrative systematic review
Source: J Cancer Surviv. 2024 Mar 23;19(4):1357–84. doi: 10.1007/s11764-024-01558-7 (PMC12283766; doi:10.1007/s11764-024-01558-7)
Supplement: Supplementary file 1 — Supplementary file1 (DOCX 16 KB) [file 11764_2024_1558_MOESM1_ESM.docx]

**Supplementary Table 1**

**Search strategy**

Seven databases were searched on 20 December 2022 to identify relevant studies (APA PsycINFO (EBSCOhost), CINAHL (EBSCOhost), Cochrane CENTRAL Register of Controlled Trials), Google Scholar, Medline (EBSCOhost), Scopus, and Web of Science Core Collection. For Google Scholar, only the first 50 search results were considered. Searches returned a total of 1,843 results. Search terms and number of search results by database:

**APA PsycINFO via EBSCOhost (5)**

(("non muscle invasive bladder cancer" OR NMIBC) AND (bereave* OR caregiver* OR cognitive OR “daily-living” OR decision* OR diet* OR domestic OR emotion* OR employment OR “end-of-life” OR exercise* OR existential OR family OR fear OR finance* OR “health care” OR hospice OR housekeeping OR information* OR interperson* OR intima* OR isolat* OR lonel* OR mental* OR nutrition* OR pain* OR palliative OR partner* OR “patient-clinician” OR physical OR practical OR psychological OR psychosocial OR "quality of life" OR sadness OR social* OR spiritual* OR spous* OR terminal OR transition*) AND (care* OR need* OR require* OR support* OR want*))

**CINAHL via EBSCOhost (109)**

(("non muscle invasive bladder cancer" OR NMIBC OR ((MH "Bladder Neoplasms") AND "non muscle invasive")) AND (bereave* OR caregiver* OR cognitive OR “daily-living” OR decision* OR diet* OR domestic OR emotion* OR employment OR “end-of-life” OR exercise* OR existential OR family OR fear OR finance* OR “health care” OR hospice OR housekeeping OR information* OR interperson* OR intima* OR isolat* OR lonel* OR mental* OR nutrition* OR pain* OR palliative OR partner* OR “patient-clinician” OR physical OR practical OR psychological OR psychosocial OR "quality of life" OR sadness OR social* OR spiritual* OR spous* OR terminal OR transition*) AND (care* OR need* OR require* OR support* OR want*))

**Cochrane Central Register of Controlled Trials (291)**

| #1 | ("non muscle invasive bladder cancer" OR NMIBC):ti,ab,kw |
| --- | --- |
| #2 | MeSH descriptor: [Urinary Bladder Neoplasms] explode all trees |
| #3 | ("non muscle invasive"):ti,ab,kw |
| #4 | #2 AND #3 |
| #5 | #1 OR #4 |
| #6 | (bereave* OR caregiver* OR cognitive OR “daily-living” OR decision* OR diet* OR domestic OR emotion* OR employment OR “end-of-life” OR exercise* OR existential OR family OR fear OR finance* OR “health care” OR hospice OR housekeeping OR information* OR interperson* OR intima* OR isolat* OR lonel* OR mental* OR nutrition* OR pain* OR palliative OR partner* OR “patient-clinician” OR physical OR practical OR psychological OR psychosocial OR "quality of life" OR sadness OR social* OR spiritual* OR spous* OR terminal OR transition*):ti,ab,kw |
| #7 | (care* OR need* OR require* OR support* OR want*):ti,ab,kw |
| #8 | #5 AND #6 AND #7 |

**Google Scholar (50)**

"non muscle invasive bladder cancer" AND supportive care

**MEDLINE via EBSCOhost (605)**

(("non muscle invasive bladder cancer" OR NMIBC OR ((MH "Urinary Bladder Neoplasms") AND "non muscle invasive")) AND (bereave* OR caregiver* OR cognitive OR “daily-living” OR decision* OR diet* OR domestic OR emotion* OR employment OR “end-of-life” OR exercise* OR existential OR family OR fear OR finance* OR “health care” OR hospice OR housekeeping OR information* OR interperson* OR intima* OR isolat* OR lonel* OR mental* OR nutrition* OR pain* OR palliative OR partner* OR “patient-clinician” OR physical OR practical OR psychological OR psychosocial OR "quality of life" OR sadness OR social* OR spiritual* OR spous* OR terminal OR transition*) AND (care* OR need* OR require* OR support* OR want*))

**Scopus (314)**

(("non muscle invasive bladder cancer" OR NMIBC) AND (bereave* OR caregiver* OR cognitive OR “daily-living” OR decision* OR diet* OR domestic OR emotion* OR employment OR “end-of-life” OR exercise* OR existential OR family OR fear OR finance* OR “health care” OR hospice OR housekeeping OR information* OR interperson* OR intima* OR isolat* OR lonel* OR mental* OR nutrition* OR pain* OR palliative OR partner* OR “patient-clinician” OR physical OR practical OR psychological OR psychosocial OR "quality of life" OR sadness OR social* OR spiritual* OR spous* OR terminal OR transition*) AND (care* OR need* OR require* OR support* OR want*))

**Web of Science Core Collection (469)**

(("non muscle invasive bladder cancer" OR NMIBC) AND (bereave* OR caregiver* OR cognitive OR “daily-living” OR decision* OR diet* OR domestic OR emotion* OR employment OR “end-of-life” OR exercise* OR existential OR family OR fear OR finance* OR “health care” OR hospice OR housekeeping OR information* OR interperson* OR intima* OR isolat* OR lonel* OR mental* OR nutrition* OR pain* OR palliative OR partner* OR “patient-clinician” OR physical OR practical OR psychological OR psychosocial OR "quality of life" OR sadness OR social* OR spiritual* OR spous* OR terminal OR transition*) AND (care* OR need* OR require* OR support* OR want*))
