# Supplementary material for: Identifying the supportive care needs of people affected by non-muscle invasive bladder cancer: An integrative systematic review
Source: J Cancer Surviv. 2024 Mar 23;19(4):1357–84. doi: 10.1007/s11764-024-01558-7 (PMC12283766; doi:10.1007/s11764-024-01558-7)
Supplement: Supplementary file 2 — Supplementary file2 (DOCX 26 KB) [file 11764_2024_1558_MOESM2_ESM.docx]

| **Qualitative Methods** | **Item number of check list** | | | | | | |
| --- | --- | --- | --- | --- | --- | --- | --- |
|  | **S1.** | **S2.** | **1.1.** | **1.2.** | **1.3.** | **1.4.** | **1.5.** |
| Alcorn et al 2020 | Y | Y | Y | Y | Y | Y | Y |
| Garg et al 2021 | Y | Y | Y | Y | Y | Y | Y |
| **Item number check list key*:** S1. Are there clear research questions, S2. Do the collected data allow to address the research questions, 1.1. Is the qualitative approach appropriate to answer the research question, 1.2. Are the qualitative data collection methods adequate to address the research question, 1.3. Are the findings adequately derived from the data, 1.4. Is the interpretation of results sufficiently substantiated by data, 1.5. Is there coherence between qualitative data sources, collection, analysis and interpretation. | | | | | | | |
| **Quantitative Descriptive Studies** | **Item number of check list** | | | | | | |
|  | **S1.** | **S2.** | **4.1** | **4.2** | **4.3** | **4.4** | **4.5** |
| Brisbane et al 2019 | Y | Y | Y | Y | Y | Y | Y |
| Catto et al 2021 | Y | Y | Y | Y | Y | Y | Y |
| Chung et al 2019 | Y | Y | Y | U | Y | Y | Y |
| Jung et al 2019 | Y | Y | Y | Y | Y | U | Y |
| Jung et al 2022 | Y | Y | Y | Y | Y | Y | Y |
| Krajewski et al 2017 | Y | Y | Y | Y | Y | Y | Y |
| Mazur et al 2023 | Y | Y | Y | Y | Y | U | Y |
| Miyake et al 2022 | Y | Y | Y | Y | Y | Y | Y |
| Park et al 2022 | Y | Y | Y | Y | Y | U | Y |
| Richards et al 2021 | Y | Y | Y | Y | U | U | Y |
| Smith et al 2022 | Y | Y | Y | Y | Y | Y | Y |
| Van der Aa et al 2009 | Y | Y | Y | Y | Y | U | Y |
| Vaioulis et al 2020 | Y | Y | Y | Y | Y | Y | Y |
| Wei et al 2014 | Y | Y | Y | Y | Y | U | Y |
| Wildeman et al 2021 | Y | Y | Y | Y | Y | Y | Y |
| Zhang et al 2020 | Y | Y | Y | Y | Y | Y | Y |
| S1. Are there clear research questions, S2. Do the collected data allow to address the research questions, 4.1. Is the sampling strategy relevant to address the research question, 4.2. Is the sample representative of the target population, 4.3. Are the measurements appropriate, 4.4. Is the risk of non-response bias low, 4.5. Is the statistical analysis appropriate to answer the research question | | | | | | | |
| **3. Mixed Methods** | **Item number of check list** | | | | | | |
|  | **S1.** | **S2.** | **5.1** | **5.2** | **4.3.** | **4.4.** | **4.5.** |
| Koo et al 2017 | Y | Y | Y | Y | Y | Y | Y |
| Kowalkowski et al 2014 | Y | Y | Y | Y | Y | Y | Y |
| Tan et al 2020 | Y | Y | Y | Y | Y | Y | N |
| S1. Are there clear research questions, S2. Do the collected data allow to address the research questions, 5.1. Is there an adequate rationale for using a mixed methods design to address the research question, 5.2. Are the different components of the study effectively integrated to answer the research question, 5.3. Are the outputs of the integration of qualitative and quantitative components adequately interpreted, 5.4. Are divergences and inconsistencies between quantitative and qualitative results adequately addressed, 5.5. Do the different components of the study adhere to the quality criteria of each tradition of the methods involved | | | | | | | |

Three levels of assessment quality scores

| Yes (Y) |
| --- |
| Unclear (U) |
| No (N) |
